# Sexual activity satisfaction in symptomatic hip osteoarthritis patients: A cross‐sectional, national web‐based study

**DOI:** 10.1111/1756-185X.14697

**Published:** 2023-04-14

**Authors:** Kai Fu, Di Zhang, Ben R. Metcalf, Kim L. Bennell, Yuqing Zhang, Win Min Oo, Leticia A. Deveza, Sarah R. Robbins, Changqing Zhang, Nigel Arden, David J. Hunter

**Affiliations:** ^1^ Department of Orthopedic Surgery Shanghai Sixth People's Hospital Affiliated to Shanghai Jiao Tong University School of Medicine Shanghai China; ^2^ Sydney Musculoskeletal Health Kolling Institute of Medical Research, The University of Sydney Sydney New South Wales Australia; ^3^ Institute of Endemic Diseases and Key Laboratory of Trace Elements and Endemic Diseases, National Health Commission of the People's Republic of China, School of Public Health Xi'an Jiaotong University Health Science Center Xi'an Shaanxi China; ^4^ Centre for Health, Exercise and Sports Medicine, Department of Physiotherapy University of Melbourne Melbourne Victoria Australia; ^5^ Division of Rheumatology, Allergy, and Immunology Massachusetts General Hospital, Harvard School of Medicine Boston Massachusetts USA; ^6^ Nuffield Department of Orthopaedics, Rheumatology and Musculoskeletal Sciences, Botnar Research Centre University of Oxford Oxford UK

## Abstract

**Aim:**

Despite high‐interest rates in sex in people with hip osteoarthritis (OA), clinicians tend not to address sexual issues, especially in older adults. The objective of this study is to evaluate sexual activity and factors associated with sexual activity satisfaction in people with symptomatic hip OA.

**Methods:**

A cross‐sectional study was conducted among 252 participants with symptomatic hip OA in Australia. Quality of sex life was assessed using the online composite of sexual activities and positions questionnaires. A Poisson model with robust variance was used to calculate the prevalence ratio (PR). Factors that showed a univariate association with sexual satisfaction were then included in a multivariable model. PR with corresponding 95% confidence intervals (CI) are reported.

**Results:**

Among the 282 participants registered on the study website, 252 met the inclusion criteria, and 60.3% (152/252) completed the sexual activity questionnaires. Hip OA interfered with sexual activity in 70.0% of the participants. High confidence in completing sexual activity (PR: 0.53, 95% CI: 0.36 to 0.77) was associated with an increased prevalence ratio of sexual satisfaction. High anxiety, depression or stress during sexual activity (PR: 1.33, 95% CI: 1.10 to 1.60) was associated with an increased prevalence ratio of sexual dissatisfaction after adjusting for hip pain level and perceived partner's orgasm.

**Conclusion:**

Although a large proportion of people with hip OA remain sexually active, a substantial proportion of persons are dissatisfied with their sexual activity. Hip OA interfered with sexual activity in most participants. Psychological factors were found to be associated with sexual activity satisfaction.

**Key point**

Hip OA interferes with sexual activity and psychological factors are related to its satisfaction.

## 1 INTRODUCTION

Osteoarthritis (OA) is a highly prevalent and painful joint disorder which imposes a substantial individual and societal burden.^1^ Compared with healthy controls, multiple aspects of quality of life in older adults with knee or hip OA are significantly affected.^2^ One important and often ignored aspect of quality of life is sexual function and satisfaction, as this has been shown to have a strong relationship with quality of life.

Sexual health is an important part of human well‐being and sexuality is defined as “a central aspect of being human throughout life” by the World Health Organization. A previous analysis of 121 persons with hip OA indicated that two‐thirds of them had sexual difficulties due to hip pain and stiffness rather than loss of libido.^3^ Despite continued interest in sex in people with hip arthritis, clinicians tend not to address sexual issues with individuals, especially in older adults.^4^, ^5^ Neither current hip scoring systems nor self‐assessment OA questionnaires cover this issue. The avoidance of the topic and lack of data collection mean there is little information about the burden of sexual dysfunction in persons with hip OA, especially their sexual activity satisfaction, despite its importance for the quality of life.

Although sexual activity is highly important to both men and women, compared with rheumatoid arthritis, OA‐targeted research on sexual activity is rare.^6^ Most focus is on the impact of total hip arthroplasty in persons with end‐stage hip OA.^7^ There is a need to better understand this issue in persons with hip OA and to find ways to improve their quality of life. Thus the objective of this study was to evaluate sexual activity satisfaction prevalence and factors associated with sexual satisfaction in persons with symptomatic hip OA.

## 2 MATERIALS AND METHODS

### 2.1 Study design

This was a cross‐sectional analysis evaluating sexual activity dissatisfaction in people with hip OA. The data were derived from the Internet‐based hip osteoarthritis pain exacerbation (iHOAP) study and recruitment did not focus on sexual function as a criterion or outcome. The study was approved by the ethics committees of The University of Sydney (Human Research Ethics Committee [HREC] 2014/801) and the University of Melbourne (HREC 1443509), and all participants provided informed consent. Eligible participants were required to log on to the study website to complete the questionnaires designed for sexual activity assessment. Patients or members of the public were not involved in the design, or conduct, or reporting, or dissemination plans of this study.

### 2.2 Participants

To be eligible for inclusion, participants were required to: be ≥40 years old; have hip pain on most days (5–7 d/wk or 20–30 d/m); have at least one hip meeting American College of Rheumatology criteria for hip OA;^8^ have a Kellgren and Lawrence (KL) grade of hip OA ≥2;^9^ have an active email account and access to the internet and computer; and have a good understanding of spoken and written English. Participants were excluded if they had a history of total hip replacement in the index hip, a scheduled total hip replacement or a scheduled consultation with an orthopedic surgeon to have a total hip replacement of the symptomatic hip(s), a history of inflammatory arthritis, osteonecrosis or Paget's disease affecting the hip.

### 2.3 Data collection

An online screening survey tool was designed to recruit eligible study participants. We collected sociodemographic data, including age, gender, body mass index (BMI), Australian residential state and education level. The Hip Disability and Osteoarthritis Outcome Score (HOOS) was used to assess participants' opinions about their hip and associated problems. This questionnaire consists of 5 subscales: Pain, Other symptoms, Function in daily living (ADL), Function in sport and recreation (Sport/Rec) and hip‐related Quality of life (QOL). A normalized score (100 indicating no symptoms and 0 indicating extreme symptoms) is calculated for each subscale. Sexual activity was assessed using a composite of sexual activity and sexual position instruments. Sexual satisfaction, sexual frequency, psychological factors and other related questions were asked.^3^, ^10^, ^11^, ^12^, ^13^, ^14^ Briefly, participants were asked to rate their current sexual satisfaction (satisfied or not) and sexual activity frequency (0, none, to 5, more than once a week). They were asked to provide information about whether hip OA interfered with their sexual relationship (0, not at all, to 3, ended sexual relationship).

**FIGURE 1:**
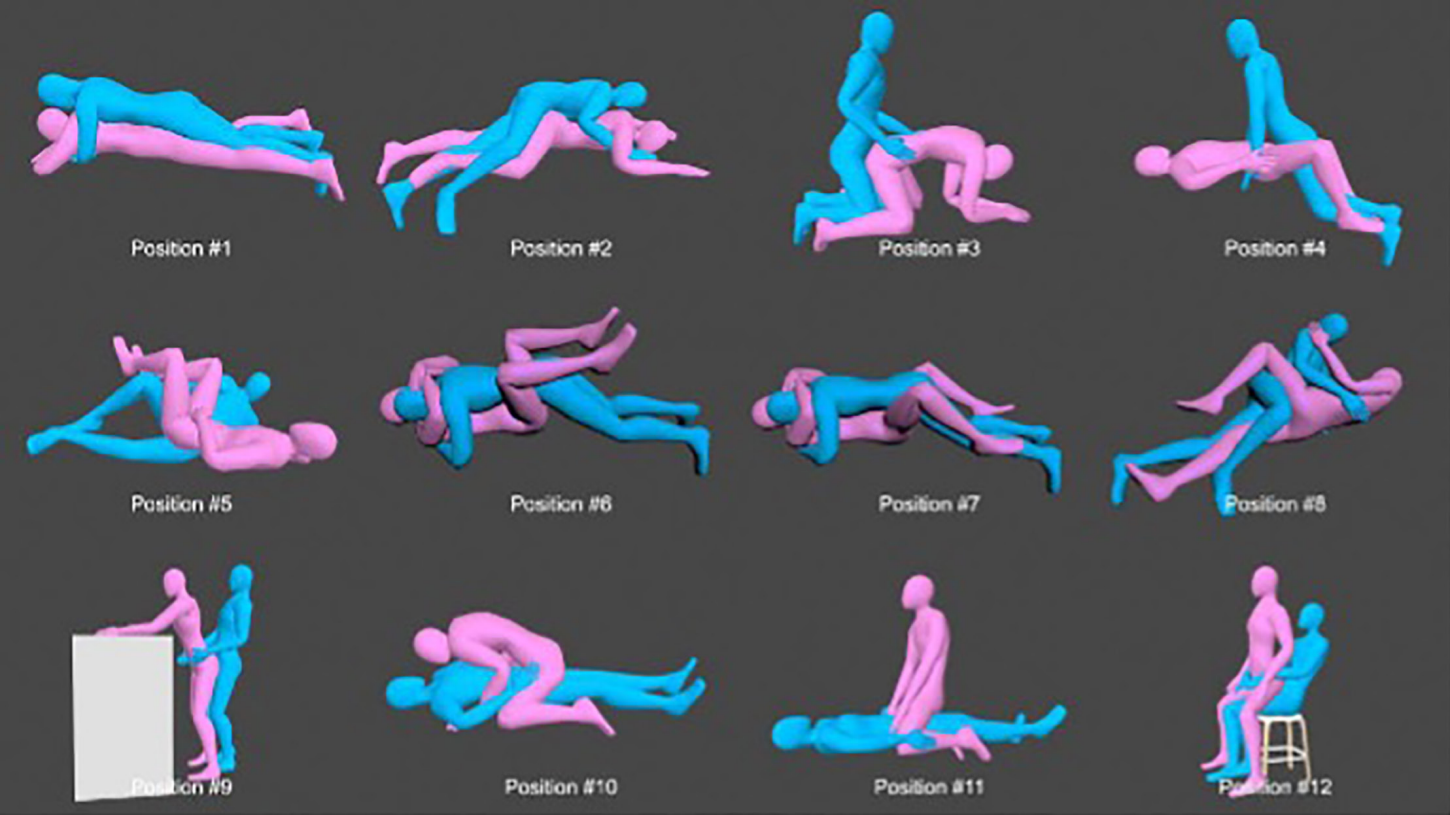
The 12 commonly used sexual positions in this study (Reference #11, permission through Elsevier).

We used the Depression, Anxiety and Stress Scale ‐ 21 Items (DASS‐21) to measure the emotional states of depression, anxiety and stress over the past week among the participants.^15^ Each of the 3 DASS‐21 scales contains 7 items. Each item is calculated on a 4‐point rating scale ranging from 0 (did not apply to me at all) to 3 (applied to me very much, or most of the time). Scores for depression, anxiety and stress were calculated by summing the scores for the relevant items. The total score of each scale was multiplied by 2 and ranged from 0 to 42, and a higher score means potentially higher severity. The recommended cut‐off scores of each dimension (depression, anxiety and stress) for conventional severity labels (normal, mild, moderate, severe and extremely severe) were used.^15^, ^16^ Schematic images of 12 common sexual positions were included in the survey. Participants were asked to report which sexual position(s) they had used during the last week^11^, ^17^ (Figure 1), and could choose 1 or more. Data were collected on a secure password‐protected study website located on a secure server.

Anteroposterior radiographs of the pelvis, including both hips, centered 5 cm on average above the pubic symphysis were taken with the participant in the supine position. KL classification was used to score the severity of hip OA. Participants were asked to provide their most recent hip radiographs, and further radiographic grading of hip OA was read by 2 rheumatologists (LAD and DJH) in our study. The inter‐rater reliability was assessed using 12 radiographs providing a weighted kappa of 0.6 for KL grades.

### 2.4 Statistical methods

Descriptive statistics were summarized as mean (SD) for continuous variables and frequency (%) for categorical variables. Participants who chose not to answer the sexual activity questionnaires were excluded from this analysis. The Poisson model with robust variance was used to calculate the prevalence ratio (PR).^18^ We explored univariate associations between demographic factors (age, gender, BMI and education level), pain level, OA radiographic severity and psychological factor (depression, anxiety or stress) that we considered plausibly associated with sexual activity dissatisfaction. Factors that showed a univariate association (*P* < 0.05) were then included in a multivariable model. PR with corresponding 95% confidence intervals (CI) are reported. Stata, version 15 (StataCorp LLC, College Station, TX, USA), was used to analyze the data.

### 2.5 Source of Funding

This work was supported by a National Health and Medical Research Council program grant (#APP631717).

## 3 RESULTS

Of the 252 participants registered on the study website, 160 (63.5%) were willing to complete the sexual activity questionnaires. Eight people with missing data were excluded, resulting in 152 participants included in the final analysis. The included participants (average age: 60.9 [SD: 9.6] years) were younger than the excluded (average age: 63.6 years). Included participants had a BMI of 28.6 (SD: 5.9) kg/m^2^, and 115 (75.7%) were female. The females (average age: 60.4 years) were younger than the males (average age: 64.3 years). Most participants were from the most populous Australian states of New South Wales, Victoria and Queensland. More than 60% of the participants had obtained a tertiary qualification. The average HOOS score for each subscale is shown in Table 1. More than half (58.6%) of the participants had KL grade 3 of their index hip. (Table 1).

**TABLE 1:** Characteristics of the study population.

Characteristic	Excluded (*n* = 100)	Included (*n* = 152)	Female (*n* = 115)	Male (*n* = 37)
Age, y^a^	63.6 (SD: 8.1)	60.9 (SD: 9.6)	60.4 (SD: 8.1)	64.3 (SD: 8.4)
BMI (kg/m^2^)	28.8 (SD: 6.3)	28.5 (SD: 5.9)	28.3 (SD: 5.8)	29.6 (SD:6.1)
State				
New South Wales	35 (35%)	58 (38.2%)	45 (39.1%)	13 (35.1%)
Victoria	31 (31.0%)	31 (20.4%)	21 (18.3%)	10 (27.0%)
Queensland	18 (18.0%)	42 (27.6%)	32 (27.8%)	10 (27.0%)
Other state/ territory	16 (16.0%)	21 (13.8%)	7 (14.8%)	4 (10.9%)
Education level				
Less than high school	19 (19.0%)	18 (11.8%)	16 (13.9%)	2 (5.4%)
Completed high school	20 (20.0%)	38 (25.0%)	24 (20.9%)	14 (37.8%)
Tertiary qualification	61 (61.0%)	96 (63.2%)	75 (65.2%)	21 (56.8%)
Hip Disability and Osteoarthritis Outcome Score				
Pain	56.9 (SD: 19.1)	54.6 (SD: 16.0)	54.7 (SD: 16.3)	54.1 (SD: 15.3)
Symptoms	55.3 (SD: 19.9)	51.5 (SD: 19.0)	50.5 (SD: 18.0)	54.7 (SD: 21.5)
ADL	59.8 (SD: 23.1)	59.2 (SD: 20.9)	59.8 (SD: 20.7)	57.3 (SD: 21.4)
Sport/Recreation	38.5 (SD: 28.2)	35.2 (SD: 26.3)	34.7 (SD: 26.3)	36.7 (SD: 26.5)
QOL	43.9 (SD: 21.4)	40.0 (SD: 18.3)	39.6 (SD: 18.7)	40.7 (SD: 17.0)
KL grade (≥ 2)				
2	34 (34%)	44 (28.9%)	36 (31.3%)	8 (21.6%)
3	53 (53%)	89 (58.6%)	67 (58.3%)	22 (59.5%)
4	7 (7%)	19 (12.5%)	12 (10.4%)	7 (18.9%)

Abbreviations: ADL, activities of daily living; BMI, body mass index; SD, standard deviation; IQR, interquartile range; KL, Kellgren and Lawrence QOL, quality of life.

^a^ Excluded vs included: *P* = 0.021; female vs male *P* = 0.014.

### 3.1 Sexual satisfaction and sexual activity

As shown in Table 2, around 25.0% (38/152) of participants were dissatisfied with their current sexual activity quality. When rating their perceived sexual satisfaction, 23.6% (36/152) of the participants thought their partner was dissatisfied, but with a high rate of the partner's orgasm. The majority (53.0%) of the females rated their partner as always having an orgasm during sexual activity while only 16.2% of the males rated their partner as always having an orgasm. Forty‐one percent (63/152) of the participants had sexual activity frequency of less than once/mo. For psychological factors during sexual activity, the median score for anxiety, depression or stress was 4 (interquartile range [IQR] 2; range 1, always, to 5, never), and the median of confidence in completing sexual activity was 3 (IQR 2; range 1, very low, to 5, very high).

**TABLE 2:** Sexual activity, sexual satisfaction, and depression, anxiety and stress (Depression, Anxiety and Stress Scale – 21 Items/DASS‐21).

Variable	Total (*n* = 152)	Female (*n* = 115)	Male (*n* = 37)
Sexual satisfaction			
Unsatisfied	38 (25%)	28 (24.3%)	10 (27%)
Satisfied	114 (75%)	87 (75.7%)	27 (73%)
Perceived sexual satisfaction			
Unsatisfied	36 (23.6%)	27 (23.5%)	9 (24.3%)
Satisfied	116 (76.4%)	88 (76.5%)	28 (75.7%)
Partner orgasm			
Always	67 (44.1%)	61 (53.0%)	6 (16.2%)
Often	51 (33.6%)	37 (32.2%)	14 (37.8%)
Half of the time	17 (11.2%)	9 (7.8%)	8 (21.6%)
Seldom	11 (7.2%)	5 (4.3%)	6 (16.2%)
Almost never	6 (3.9%)	3 (2.6%)	3 (8.1%)
Frequency of sexual activity			
≥ once/wk	33 (21.7%)	26 (22.6%)	7 (27.0%)
≥ once/2–3 wk	31 (20.4%)	22 (19.1%)	9 (24.3%)
≥ once/mo	25 (16.4%)	16 (13.9%)	9 (24.3%)
≥ once/2–3 mo	19 (12.5%)	18 (15.7%)	1 (2.7%)
≥ once/6–12 mo	44 (28.9%)	33 (28.7%)	11 (29.7%)
DASS‐21			
Depression			
Normal	122 (80.3%)	94 (81.7%)	28 (75.7%)
Mild	12 (7.9%)	10 (8.7%)	2 (5.4%)
Moderate	9 (5.9%)	6 (5.2%)	3 (8.1%)
Severe	4 (2.6%)	2 (1.7%)	2 (5.4%)
Extremely severe	5 (3.3%)	3 (2.6%)	2 (5.4%)
Anxiety			
Normal	130 (85.5%)	97 (84.3%)	33 (89.2%)
Mild	5 (3.3%)	5 (4.3%)	0
Moderate	13 (8.6%)	10 (8.7%)	3 (8.1%)
Severe	0	0	0
Extremely severe	4 (2.6%)	3 (2.6%)	1 (2.7%)
Stress			
Normal	132 (86.8%)	100 (87.0%)	32 (86.5%)
Mild	12 (7.9%)	10 (8.7%)	2 (5.4%)
Moderate	6 (3.9%)	4 (3.5%)	2 (5.4%)
Severe	2 (1.3%)	1 (0.9%)	1 (2.7%)
Extremely severe	0	0	0

Among the 12 different sexual positions, positions #4 (74/152, 48.7%), #6 (74/152, 48.7%) and #7 (73/152, 48.0%) were the most commonly used by participants (Table 3). However, more than half of the males preferred female dominant sexual positions of #10 (21/37, 56.8%) and #11 (19/37, 51.4%).

**TABLE 3:** Distribution of the 12 commonly used sexual positions.

Position	Total (*n* = 152)	Female (*n* = 115)	Male (*n* = 37)
#1	27 (17.8%)	19 (16.5%)	8 (21.6%)
#2	29 (19.0%)	23 (20%)	6 (16.2%)
#3	51 (33.5%)	36 (31.3%)	15 (40.5%)
#4	74 (48.7%)	55 (47.8%)	19 (51.4%)
#5	49 (32.3%)	39 (33.9%)	10 (27.0%)
#6	74 (48.7%)	56 (48.7%)	18 (48.6%)
#7	73 (48%)	52 (45.2%)	21 (56.8%)
#8	39 (25.6%)	25 (21.7%)	14 (37.8%)
#9	25 (16.4%)	8 (7.0%)	17 (45.9%)
#10	43 (28.3%)	22 (19.1%)	21 (56.8%)
#11	47 (31%)	28 (24.3%)	19 (51.4%)
#12	9 (5.9%)	6 (5.2%)	3 (8.1%)

### 3.2 Hip OA and sexual relationship

Most participants (110, 72.4%) felt that their hip OA influenced their sexual activity, including 9.1% who ended sexual relationships because of the condition. Only 19.1% of females reported that hip OA did not influence sexual activity at all, while nearly half (47.6%) of the males reported the same (Table 4). When dealing with sexual problems, only 38.2% of participants would like to discuss this issue with a family doctor or a specialist in the hospital. More females (40.0%) would search for material for this particular problem than males (21.6%). The main reason was that most participants considered the topic too personal (Table 4).

**TABLE 4:** Hip osteoarthritis (OA) and sexual relationship.

Issues related to sexual activity	Total (*n* = 152)	Female (*n* = 115)	Male (*n* = 37)
Influence of hip OA on sexual activity			
Not at all	42 (27.3%)	22 (19.1%)	20 (47.6%)
Slightly	58 (38.3%)	47 (30.9%)	11 (19.0%)
Considerably	39 (25.3%)	34 (22.4%)	5 (12.8%)
Ended sexual relationship	13 (9.1%)	12 (7.9%)	1 (7.7%)
Preferred advice seeking^a^			
Discussion with family doctor	38 (25%)	30 (26.1%)	8 (21.6%)
Discussion with hospital doctor	20 (13.2%)	16 (13.9%)	4 (10.8%)
Discussion with medical social worker	19 (12.5%)	15 (13.0%)	4 (10.8%)
Material for this particular problem	54 (35.5%)	46 (40%)	8 (21.6%)
Reason for not discussing with doctor			
Doctor was too busy in the clinic	26 (17.1%)	19 (16.5%)	7 (18.9%)
Asked, but could not obtain appropriate information	12 (7.9%)	10 (8.7%)	2 (5.4%)
Topic was too personal	114 (75%)	86 (74.8%)	28 (75.7%)

^a^ Participants can choose more than 1 or none.

### 3.3 Factors associated with sexual dissatisfaction

To determine factors associated with sexual satisfaction, the univariable analysis was performed with different variables. HOOS pain, symptoms, ADL, Sports/Rec, and QOL were inversely associated with sexual dissatisfaction in the univariable analyses, while X‐ray scoring was not associated with sexual satisfaction. (Table 5). Anxiety, depression or stress during sexual activity, and confidence in completing sexual activity were found to be statistically significantly associated with sexual dissatisfaction in univariate analyses. When these variables were included in a multivariable model, high confidence in completing sexual activity (PR: 0.53, 95% CI: 0.36 to 0.77) was associated with an increased prevalence ratio of sexual satisfaction. High anxiety, depression or stress during sexual activity (PR: 1.33, 95% CI: 1.10 to 1.60) was associated with an increased prevalence ratio of sexual dissatisfaction (Table 5).

**TABLE 5:** Factors associated with sexual dissatisfaction in 152 participants with symptomatic hip osteoarthritis using Poisson regression models with robust variance.

Variable	Univariable analysis		Multivariable analysis	
	Prevalence ratio (95% CI)	*P* value	Prevalence ratio (95% CI)	*P* value
Age	1.01 (0.98, 1.04)	0.356		
Gender^a^	0.90 (0.48, 1.68)	0.742		
BMI	1.02 (0.99, 1.07)	0.205		
Education level	0.97 (0.66, 1.43)	0.891		
Hip Disability and Osteoarthritis Outcome Score				
Pain	0.98 (0.96, 0.99)	**0.034**	1.00 (0.97, 1.04)	0.829
Symptoms	0.98 (0.97, 0.99)	**0.025**	0.99 (0.96, 1.02)	0.524
ADL	0.98 (0.97, 0.99)	**0.002**	1.02 (0.99, 1.05)	0.111
Sport/Recreation	0.98 (0.97, 0.99)	**0.007**	0.99 (0.98, 1.00)	0.147
QOL	0.98 (0.97, 0.99)	**0.008**	0.99 (0.97, 1.00)	0.199
KL grade	1.16 (0.74, 1.82)	0.505		
Anxiety, depression or stress	1.77 (1.47, 2.13)	**0.001**	1.33 (1.10, 1.60)	**0.003**
Confidence	0.46 (0.36, 0.59)	**<0.001**	0.53 (0.36, 0.77)	**0.001**

^a^ Male was used as reference.

Abbreviations: ADL, activities of daily living; BMI, body mass index; SD, standard deviation; IQR, interquartile range; KL, Kellgren and Lawrence; QOL, quality of life.

## 4 DISCUSSION

In this study, we found a substantial proportion of individuals with symptomatic hip OA were sexually active but were dissatisfied with their sexual activity. Most participants considered that hip OA influenced their sexual activity to some extent, but a gender difference was apparent. Psychological factors, including anxiety, depression or stress during sexual activity and lower confidence in completing sexual activity, were significantly associated with sexual dissatisfaction.

Consistent with limited previous studies, we found a large proportion of people with symptomatic hip OA remain sexually active.^4^ Although recognized as a fundamental driving force, the topic of sexuality is often misunderstood or neglected in older adults. Studies focused on factors associated with sexual activity in adults over 60 years old showed that partner‐related factors, erectile function and past active sexuality were important determinants of being sexually active.^19^ Given the prevalence of hip OA and until now, the largely unreported impact of hip OA on sexual activity, this may need to be added to this list.

Although most participants with hip OA remain sexually active, the satisfaction level of sexual activity varies compared with other populations. In our study, we found that around 25% of the participants were dissatisfied with their sexual activity. However, another study performed on 474 normal Australian females aged 40–80 years, showed that only 2.5% of women reported low sexual relationship satisfaction.^20^ Huang et al^21^ reported that sexual desire or interest in 1977 participants aged 45 to 80 years was significantly associated with the overall level of sexual satisfaction, indicating the higher desire or interest, the higher sexual satisfaction. Consistent with our findings, 60% of the female participants reported some sexual activity in the previous 3 months.

Many factors can contribute to sexual problems, which may lead to varied satisfaction levels. One study conducted in Australia showed that sex remains important to older men and the potentially modifiable risk factors affecting sexual activity were diabetes, depression and medication use.^22^ For most older men, androgen deficiency is not the major cause of sexual problems but modifiable risk factors like chronic disease, depression and insomnia.^23^ Depressed mood was associated with certain sexual difficulties but not with impotence in 169 persons with rheumatic diseases, including OA.^24^

More than 70% of the participants in our study considered that hip OA interfered with their sexual activity. Consistently, Carlos et al found that hip arthritis affected sexual activity in around 80% of sexually active patients.^4^ People with hip OA in our study preferred sexual positions in which they could minimize the range of motion of the hip joint to avoid the extremes of hip flexion, adduction, and internal rotation.^11^, ^17^ Although sexual activity was influenced by hip OA in a large proportion of our participants, the majority (more than 70%) were still satisfied with their sexual activity. This unparalleled relationship was further confirmed by the non‐associated relationship between hip OA radiographic severity and sexual activity satisfaction. Our study found no association between X‐ray scoring and sexual satisfaction, indicating that X‐ray scoring is a poor predictor of QOL. Instead, psychological factors during sexual activity like anxiety, depression, stress levels or confidence in completing sexual activity, play dominant roles in sexual activity satisfaction in people with symptomatic hip OA.

Although pain, stiffness and fatigue associated with hip OA may make physical intimacy difficult, these symptoms may be ameliorated during sexual activity by good communication between partners and timing medication use.^6^ It is also advisable to practice different sexual positions; using joint supports can help to maintain sexual posture, and a hot bath beforehand is also recommended to relax the muscles.^25^ In severe cases, joint replacement is possible, and most patients report an improved sex life.^13^ In addition, appropriate psychotherapy and counseling, where necessary, may have a positive impact. People may need to be taught to be creative and willing to experiment and learn to relax in response, which can enhance the sexual experience for people with hip OA.^6^

Today, internet use is an integral part of everyday life. According to the Australian Bureau of Statistics, active internet users represented 89% and 88% of the total Australian population in 2015 and 2016.^26^ Studies have shown that web‐based surveys and data collected through web‐based methods are of consistent or even better quality than those collected through traditional methods.^27^, ^28^ Therefore, web questionnaires are nowadays the most employed survey mode in quantitative research worldwide.^29^ We confirm that these instruments in this study were validated measures which have been used in previous research studies and applied to the web‐based questionnaire. We used a composite of validated sexual activities and sexual position instruments to assess the quality of sex life of our participants, and the images of sexual positions were posted on our study website for participants to report. In addition, because the topics discussed are more private, logging on to the website gives more accurate results than attending an office meeting.

Some limitations of our study need to be addressed. Participants were required to have access to the internet and a good understanding of English, meaning that the findings may not generalize to all persons with hip OA. We should acknowledge that this is not a random sample and not every recruited participant wanted to complete the questionnaires. This might have biased the sample toward those who were more satisfied with their sexual activity. In addition, we should also acknowledge that there is an educational bias. Research has found that education level and socio‐economic status are associated with erotic inequality, and there is a strong correlation between socio‐economic status and educational attainment, with people of high socio‐economic status and education tending to be more likely to use online tools, and vice versa, limiting their use.^30^ We selected variables from the univariate analysis for entry into the multivariable model rather than entering them together. While this might miss some variables, it can avoid collinearity issues between variables.^31^ We did not include the sexual activity frequency and the partner's satisfaction in the analysis, so we used perceived sexual satisfaction to describe it. We assumed that if a participant were more satisfied with their sexual activity, they would be more likely to be sexually active and rate their partner satisfied. Some other factors, such as other comorbidities or medication use, may affect sexual activity but were not included in our study. In addition, we did not collect information on relationship status in this study, and we will refine this information in future surveys.

## 5 CONCLUSION

In conclusion, our findings showed that although a large proportion of persons with hip OA remain sexually active, there is still a substantial proportion who are dissatisfied with their sexual activity and who report that their hip OA interferes with their sexual activity. The factors associated with sexual activity dissatisfaction in this sample were anxiety, depression or stress during sexual activity and confidence in completing sexual activity. These findings have several implications for research and clinical practice. Disease‐specific instruments to assess the QOL of persons with hip OA should include the impact of the condition on sexual activity. In clinical practice, health professionals should ask patients whether they would like to discuss the effect of their hip OA on their sexual activity in a sensitive manner. Education for patients with hip OA should also cover the issue of sexual activity.

## AUTHOR CONTRIBUTION

All authors made contribution to the design of the research and interpretation of the data. Conceptualization: D.J.H., K.F., D.Z.; clinical investigation: K.F., D.Z., B.R.M., K.L.B., L.A.D. and D.J.H.; writing–original draft preparation: K.F., D.Z.; writing–review and editing: B.R.M., K.L.B., Y.Z., W.M.O., L.A.D., S.R.R., C.Z., N.A. and D.J.H.; funding acquisition: D.J.H. All authors have read and agreed to the published version of the manuscript.

## CONFLICTS OF INTEREST STATEMENT

The authors report no conflicts of interest in this work.

## Data Availability

Data availability statement: The data used in this study are available from the corresponding author on request.
